# Dr. Johannes A. Jones

**Published:** 1874-05

**Authors:** 


THE CERTIFICATS OF IMPOSTORS.—*Dr. J. J. G., of Ga.*—We have so often stated that the certificates and references of traveling charlatans are mere delusions, that it is hardly worth while repeating that "Dr. Johannes A. Jones," now traveling through the South, is but one of the gang, and his Philadelphia references are not worth a straw.—*Medical and Surgical Reporter.*

